# Targeted Overexpression of Amelotin Disrupts the Microstructure of Dental Enamel

**DOI:** 10.1371/journal.pone.0035200

**Published:** 2012-04-23

**Authors:** Rodrigo S. Lacruz, Yohei Nakayama, James Holcroft, Van Nguyen, Eszter Somogyi-Ganss, Malcolm L. Snead, Shane N. White, Michael L. Paine, Bernhard Ganss

**Affiliations:** 1 School of Dentistry, Center for Craniofacial Molecular Biology, University of Southern California, Los Angeles, California, United States of America; 2 Matrix Dynamics Group, Faculty of Dentistry, University of Toronto, Toronto, Ontario, Canada; 3 School of Dentistry, University of California Los Angeles, Los Angeles, California, United States of America; Ecole Normale Supérieure de Lyon, France

## Abstract

We have previously identified amelotin (AMTN) as a novel protein expressed predominantly during the late stages of dental enamel formation, but its role during amelogenesis remains to be determined. In this study we generated transgenic mice that produce AMTN under the amelogenin (*Amel*) gene promoter to study the effect of AMTN overexpression on enamel formation in vivo. The specific overexpression of AMTN in secretory stage ameloblasts was confirmed by Western blot and immunohistochemistry. The gross histological appearance of ameloblasts or supporting cellular structures as well as the expression of the enamel proteins amelogenin (AMEL) and ameloblastin (AMBN) was not altered by AMTN overexpression, suggesting that protein production, processing and secretion occurred normally in transgenic mice. The expression of Odontogenic, Ameloblast-Associated (ODAM) was slightly increased in secretory stage ameloblasts of transgenic animals. The enamel in AMTN-overexpressing mice was much thinner and displayed a highly irregular surface structure compared to wild type littermates. Teeth of transgenic animals underwent rapid attrition due to the brittleness of the enamel layer. The microstructure of enamel, normally a highly ordered arrangement of hydroxyapatite crystals, was completely disorganized. Tomes' process, the hallmark of secretory stage ameloblasts, did not form in transgenic mice. Collectively our data demonstrate that the overexpression of amelotin has a profound effect on enamel structure by disrupting the formation of Tomes' process and the orderly growth of enamel prisms.

## Introduction

Dental enamel is the hardest tissue in vertebrates. If properly formed and cared for it is, unlike synthetic restorative materials, designed to last a lifetime under immense mechanical stress, in spite of constant challenges within the oral cavity through changes in temperature, pH, and exposure to aggressive cariogenic microorganisms. The formation of dental enamel is a prototype of functional organ development through a biomineralization process. The three main structural proteins of the forming enamel, the most abundant amelogenin (AMEL), as well as ameloblastin (AMBN) and enamelin (ENAM), are collectively referred to here as enamel matrix proteins (EMPs). The EMPs are produced at their highest levels by ameloblasts during the secretory and transition stages of amelogenesis and collectively orchestrate the proper assembly and growth of crystals within the mineralized enamel. The proteins are almost completely degraded by specific proteases such as MMP-20 mainly during the secretory/transition stage and KLK4 mainly during the transition/maturation stage, respectively, resulting in a highly ordered and purposefully designed meshwork of carbonated hydroxyapatite crystals with astonishing mechanical properties [1].

Amelotin (AMTN) is a recently discovered enamel protein [2] with very limited sequence similarity to the EMPs, although there is evidence that AMTN and other ameloblast-expressed genes, as well as secreted calcium-binding phosphoproteins (SCPPs), have evolved from a common ancestral gene [3]. *Amtn* mRNA expression is transient in ameloblasts of rodent molars from postnatal day 2 to the time of tooth eruption approximately 14 days later, but persists in the continuously erupting incisors. A detailed expression profiling of AMTN protein [4] has shown that its expression is dramatically upregulated during the transition stage in ameloblasts and continues throughout the maturation stage in a fashion similar to another recently described ameloblast gene, *Odam/Apin* [5], [6]. The expression profile of *Amtn* (and *Odam*) is thus clearly distinct from that of the three classical EMPs (*Amel*, *Ambn*, *Enam*), but parallels that of *Klk4*, which codes for a protein associated with EMP degradation and mineral maturation. The AMTN protein is secreted and has been localized to a basal lamina-like layer between ameloblasts and the enamel mineral surface in incisors of rats [7] and mice [4]. Thus it has been suggested to be involved in cell attachment, control of ion and peptide transport to and from maturing enamel, or the formation of the distinct aprismatic, specialized, superficial layer referred to as “final” enamel [8], [9]. The lack of cell culture models for maturation stage ameloblasts has hampered functional studies *in vitro*, but functional studies of specific proteins, including those controlling the biomineralization process of dental enamel, have been greatly facilitated by the development of genetically engineered mice as *in vivo* gain- and loss-of-function models. Due to the confined expression of enamel genes, unlike many other genes, gene “knock-outs” have been particularly useful in revealing the specific roles of individual enamel proteins without thwarting the interpretation of experimental phenotypes by early lethality or compensation mechanisms. Conversely, gain-of-function by the tissue-specific overexpression of enamel proteins, driven by a well-characterized 2.3 kb mouse amelogenin promoter, has significantly advanced our understanding of the contribution of individual proteins and protein fragments to amelogenesis [10], [11], [12], [13], [14]. In this paper we have used the latter technology to create several lines of experimental mice that overexpress the amelotin protein in ameloblasts. The rationale for choosing this strategy was that the transgenic mice would not only produce higher levels of AMTN, but also at an earlier stage of amelogenesis, and would thus allow us to determine the effect of AMTN on enamel prism growth. We describe the resulting effects on enamel structure and mechanical properties, protein expression patterns and cellular morphology of ameloblasts.

## Results

### Production of transgenic animals

**Figure 1:**
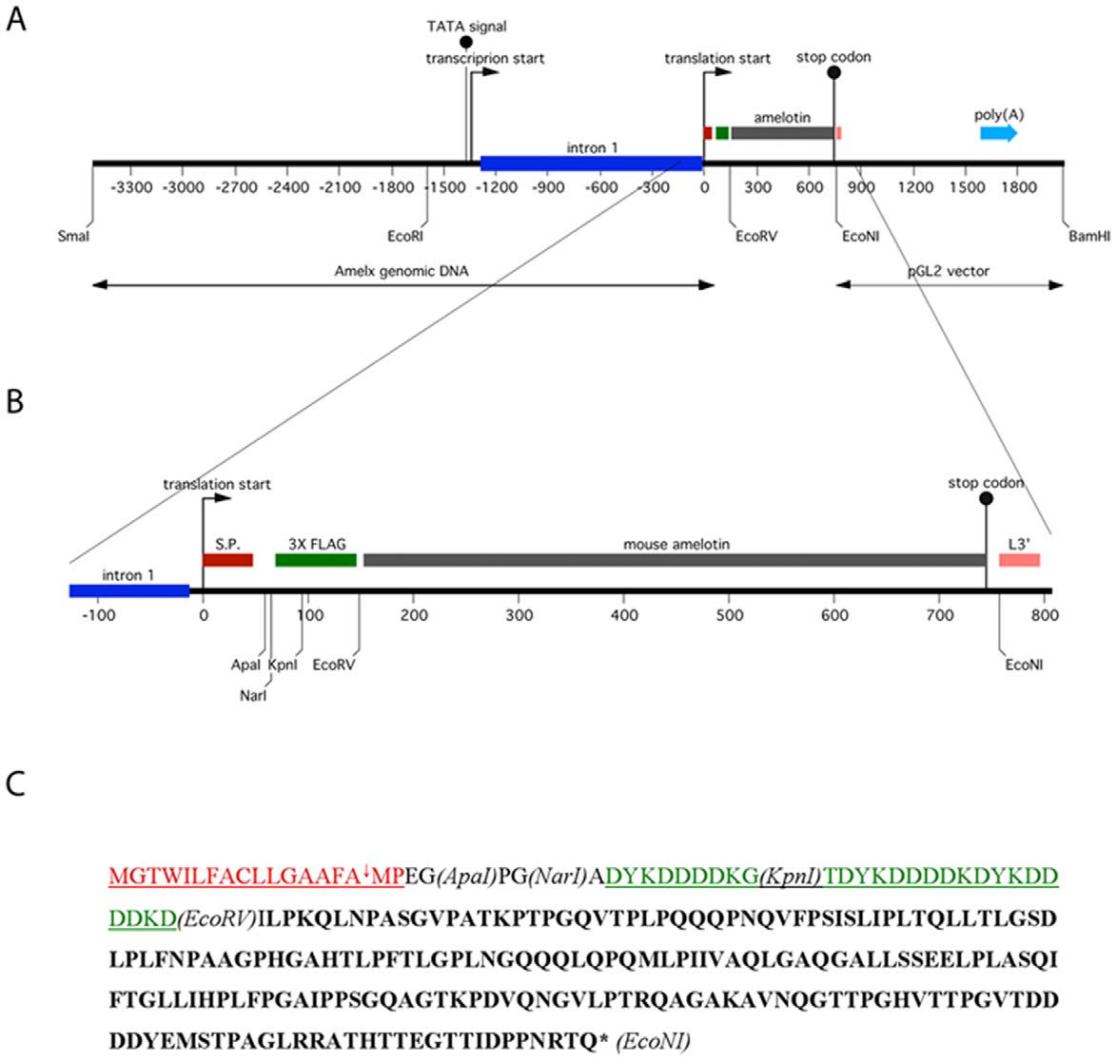
Construction and schematic representation of the amelotin transgene. (A) The 3.5 kb mouse amelogenin promoter region extending through intron 1 (blue) and the signal peptide region (red) was used to drive the triple FLAG epitope (green) containing mouse amelotin transgene construct (grey). (B) An enlarged region of the transgene's open reading frame. (C) Transgene protein sequence identifying the amelogenin signal peptide (red) and the cleavage site (arrow), the three repeats of the FLAG epitope at the amino terminus, and amelotin. The positions of relevant restriction enzyme sites are also included. Numbers refer to nucleotides with +1 being the first adenosine in the translated gene product; L3′ refers to the 3′ region of luciferase cDNA; poly(A) refers to the SV40 late poly(A) signal; S.P. refers to the amelogenin signal peptide; and *Amelx* genomic DNA refers to the mouse amelogenin gene promoter region and intron 1. The transgene was released from the vector backbone with restriction enzymes SmaI and BamHI prior to the generation of animals. The theoretical molecular weight of the unmodified transgene product is 24.4 kDa without, 26.1 kDa with signal peptide.

**Figure 2:**
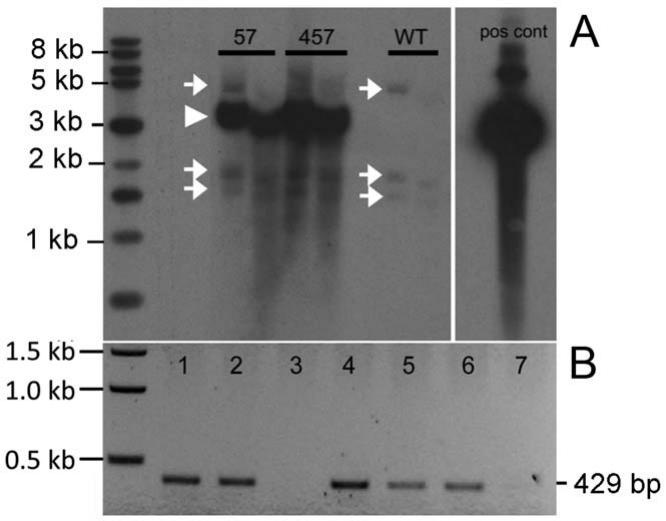
Southern Blot analysis of transgenic animals. Genomic DNA isolated from various transgenic founder lines (57, 457) and wild type (WT) littermates were digested with PstI, separated by agarose gel electrophoresis and (A) autoradiographed after hybridization to a radioactively labeled amelotin cDNA probe isolated from the EcoRV-PstI 528 bp region of the transgene cassette (see Figure 1). Three specific bands correlating to genomic DNA, each of the predicted size (4.5, 1.7 and 1.4 kb), are seen in WT samples and all transgenic animals (arrows) and serve as loading control between lanes; the arrowhead indicates the position of the transgene-specific signal at 3.115 kb correlating to a PstI-PstI transgene fragment, which is all-inclusive of the probe. (B) PCR genotyping using a forward primer contained within intron 1 and a reverse primer completely contained within the amelotin cDNA region verifies the generation of a 429 bp amplicon in transgenic mice (lanes 1, 2, 4, 5, 6), but not in non-transgenic littermates (lanes 3, 7). Samples for lanes 1 to 7 were randomly selected from litters from both transgenic lines (57 and 457).

**Figure 3:**
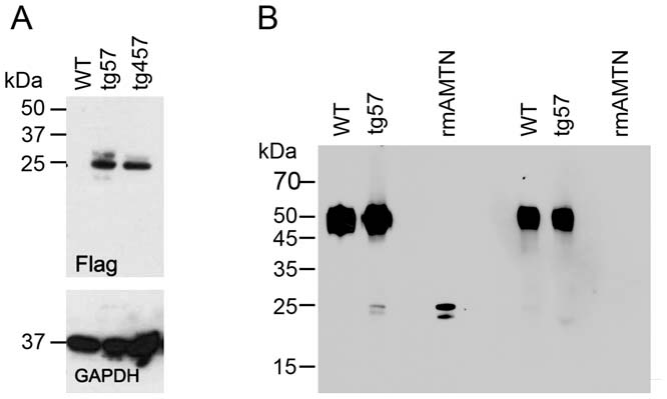
Western Blot analysis of wild type (WT) and transgenic (tg57 and tg457) 3–4-day old mice. Total protein extracts from mandibular first and second molars were probed with anti-Flag (A, upper panel) and anti-GAPDH (A, lower panel) antibodies, indicating overexpression of the transgenic protein at comparable levels in both transgenic lines. Probing extracts with an anti-amelotin antibody after enrichment via IgG immunoprecipitation (B) yielded, in addition to the IgG heavy chain band at ∼50 kDa, two immunoreactive bands at ∼24 kDa and ∼22 kDa in transgenic animals only, which was similar in size to that of bacterially expressed recombinant mouse AMTN (rmAMTN) as a positive control (B, left panel). Competition with excess rmAMTN protein abolished the 24 and 22 kDa signals in tg57 and rmAMTN, confirming specificity of the signal (B, right panel).

The vector for the site-specific overexpression of TgAMTN in transgenic mice is shown schematically in Figure 1. The hallmarks of the construct include the ∼2.3 kb murine amelogenin promoter, followed by intron 1 to allow optimal processing of the mRNA transcript, and the coding sequences for the amelogenin signal peptide to achieve efficient protein secretion into the extracellular space. Three repeats of the FLAG epitope (DYKDDDDK) were engineered into the N-terminal region of the transgene which were followed in frame by the murine amelotin coding sequence. The *Amel* promoter region was recovered from a lambda phage cDNA library, while the FLAG epitope and amelotin cDNA regions were amplified by PCR. All DNA derived from PCR were sequenced in their entirety to ensure sequence integrity of the final plasmid construct. The production of transgenic animals resulted in two independent lines (57 and 457). The presence and relative abundance of the transgene were validated and assessed by Southern blot analysis (Figure 2), confirming lines 57 and 457 as harboring similar copy numbers of the transgene (Figure 2A). Further qualitative genotyping by PCR verified the transgene status, producing a single 429 bp product only in transgenic animals, regardless of which transgenic line was analyzed (Figure 2B). Corresponding protein levels were assessed by Western blot, confirming the presence of the FLAG-tagged transgenic protein in molar tooth extracts of lines 57 and 457, but not in wild type animals at 3–4 days of age (Figure 3A). Western blot analysis with the anti-AMTN antibody FL-rmAMTN, after enrichment of the expressed AMTN protein in cell lysates by immunoprecipitation, also confirmed the presence of TgAMTN in transgenic animals of the line 57 (Fig. 3B, Amtn) and 457 (not shown), but not in wild type animals. Competition by an approximately 200-fold molar excess of recombinant murine AMTN abolished the specific signal around 24 kDa in molar tooth extracts from transgenic mice and the recombinant murine AMTN protein as positive control, but did not affect the signal of the rabbit immunoglobulin heavy chain at about 50 kDa (Fig. 3B; Amtn+Comp). Western blots with the mAMTN-1 antibody did not produce any signal (not shown).

### Immunohistochemistry

**Figure 4:**
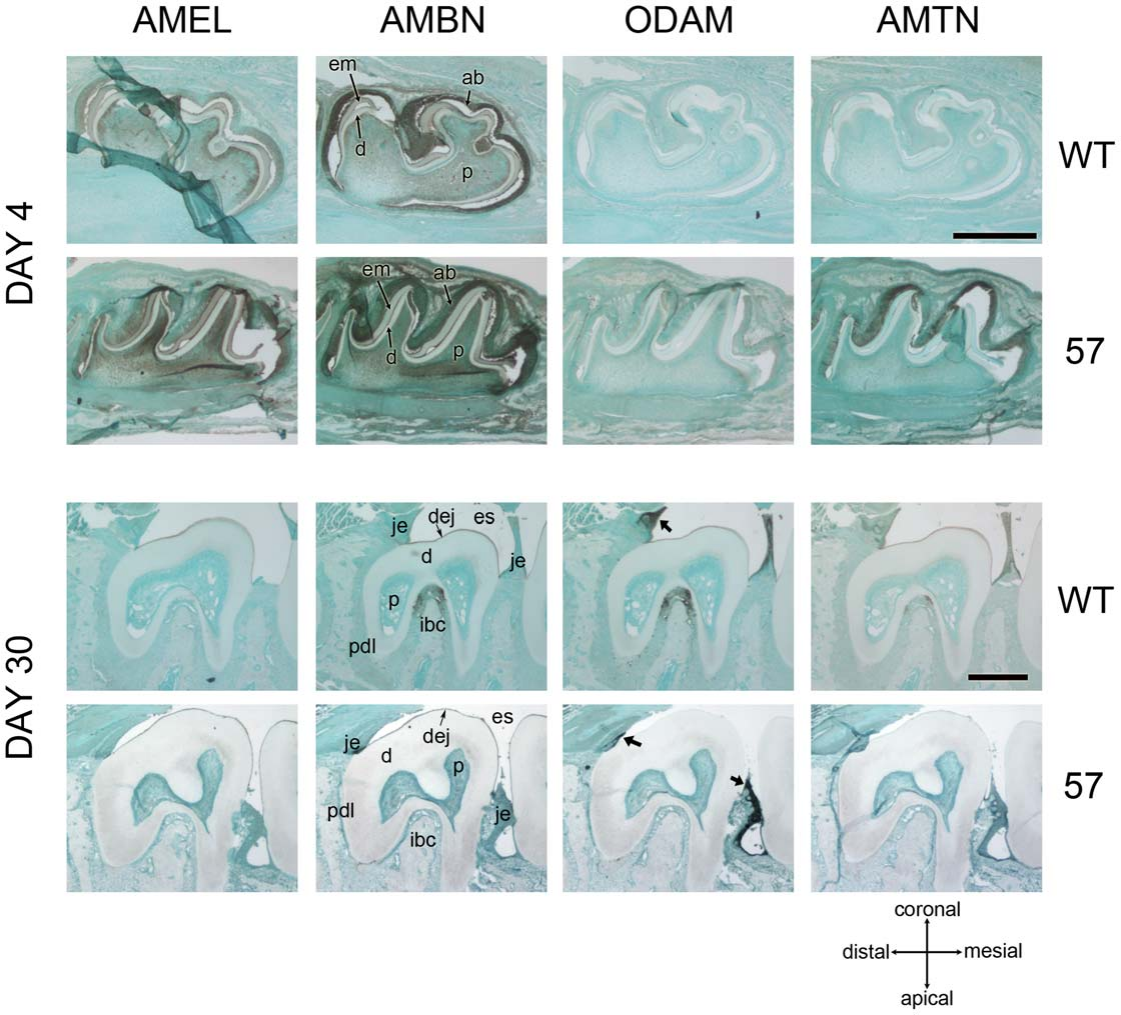
Immunohistochemical (IHC) analysis of molar teeth at pre- and post-eruptive stages. Sagittal sections of mandibular first molars at postnatal day 4 (upper panel) and second molars at day 30 (lower panel) were probed with antibodies against amelogenin (AMEL), ameloblastin (AMBN), ODAM and amelotin (AMTN) in wild type (WT) and transgenic (57) animals. Only AMTN is significantly overexpressed in transgenic animals at day 4. After tooth eruption at day 30 when the amelogenin promoter shows minimal activitythe expression levels of all proteins have returned to very low levels, except ODAM, which continues to show a signal in the junctional epithelia (arrows in lower panel). The orientation of all sections is indicated in the lower right portion. Tissue structures are indicated with the following abbreviations: ab-ameloblasts; d-dentin; dej-dentinoenamel junction; em-enamel matrix; es-enamel space; ibc-interradicular bone crest; je-junctional epithelium; p-pulp; pdl-periodontal ligament. Scale bars = 500 µm.

**Figure 5:**
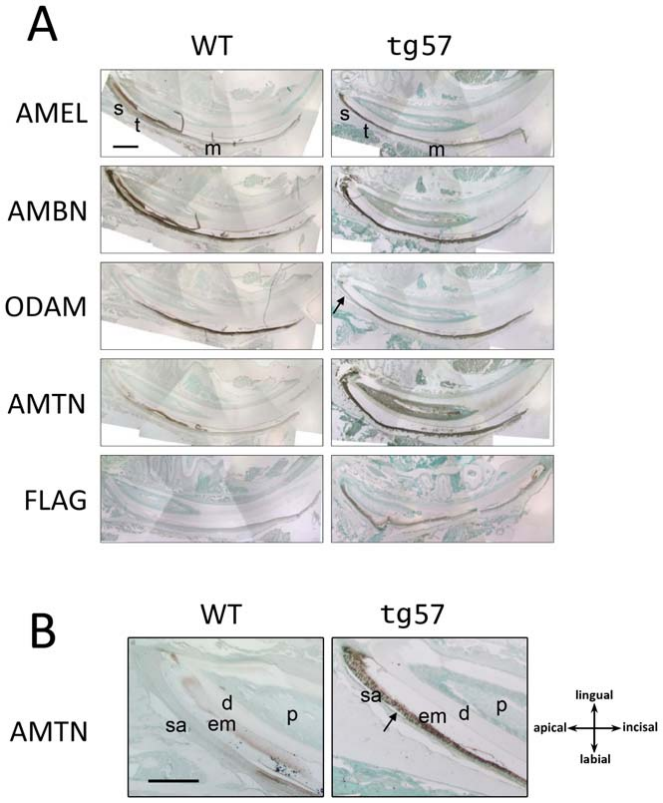
IHC analysis of mandibular incisors. (A) Sagittal sections at P30, probed with antibodies against amelogenin (AMEL), ameloblastin (AMBN), ODAM, amelotin (AMTN) and the FLAG epitope in wild type (WT) and transgenic (tg57) animals. Overexpression of AMTN in tg57 animals extends to the apical portion of the incisor. A slightly increased ODAM signal (arrow) in secretory stage ameloblasts is observed, while the signal patterns and intensities for AMEL and AMBN remained unchanged. (B) Magnified views of the apical portion of incisors, showing little expression of Amtn in wild type apical enamel tissues, but significantly increased amounts in transgenic lines. The orientation of all sections is indicated. Abbreviations: d, dentin; em, enamel matrix; m, maturation stage; p, pulp; sa, secretory stage ameloblasts; s, secretory stage; t, transition stage. Scale bars: 500 µm (A); 250 µm (B).

To verify the various levels and to determine the specific sites of TgAMTN overexpression in transgenic animals immunohistochemical analyses were conducted, which confirmed overexpression of TgAMTN in ameloblasts at the early secretory stage. Two time points, postnatal days 4 and 30 (P4 and P30), were chosen for detailed analyses in molars to reflect pre-eruptive and mature stages of tooth formation (Figure 4). The overexpression of TgAMTN in molars of transgenic line 57 was confirmed at P4, but the expression pattern and levels of other enamel proteins (AMEL, AMBN, ODAM) were not significantly altered by the early overexpression of TgAMTN. Both AMEL and AMBN showed a fairly uniform signal in ameloblasts, enamel matrix and, to a lesser extent, pulp, while ODAM was practically undetectable at this stage in all animals. At P30, where the Amel promoter driving the transgene expression is essentially inactive as seen by the lack of immunostaining for AMEL in wild type animals (Fig. 4, day 30, Amel, WT), AMBN, ODAM and AMTN produced signals in the interradicular bone crest region, possibly staining epithelial rests of Malassez. ODAM was predominantly found in the junctional epithelium, consistent with previous reports [6]. In transgenic animals (line 57) the signals for AMBN, ODAM and AMTN in the interradicular bone crest were abolished, but ODAM expression persisted in the junctional epithelium. Identical results were obtained when analyzing line 457 animals (results not shown). In the continuously erupting incisor at P30 (Fig. 5), where the entire developmental spectrum of enamel formation is present, AMTN was detected in ameloblasts from the late secretory stage to the maturation stage in wild type animals, but TgAMTN was found at higher levels and at earlier stages in the transgenic line 57 (Fig. 5A). An immunoreactive signal for the FLAG peptide was only detected in transgenic animals. The expression of AMEL, detected in wild type animals from the early secretory to the mid-maturation stage, was not altered in transgenic animals. AMBN labeling in wild type animals paralleled that of AMEL in early stage ameloblasts, but persisted farther into the maturation zone. AMBN expression was also unchanged in transgenic animals. ODAM signals in wild type animals were undetectable in apical ameloblasts, but intense in cells of the late secretory to maturation stage. In transgenic animals, the expression in late secretory to maturation stage ameloblasts was unaltered, but an ODAM signal that was slightly increased compared to wild type animals could be detected in secretory stage ameloblasts (Fig. 5A, arrow). Magnified views of the apical end of P30 incisors (Fig. 5B) illustrate the increased expression of TgAMTN in apical ameloblasts of transgenic mice at much earlier stages than in wild type animals. Notably, the pattern of TgAMTN protein expression in adjacent apical ameloblasts of line 57 animals often appeared patchy, with alternating high and low TgAMTN signal intensities in adjacent cells (Figure 5B, arrow).

### Histological analysis

**Figure 6:**
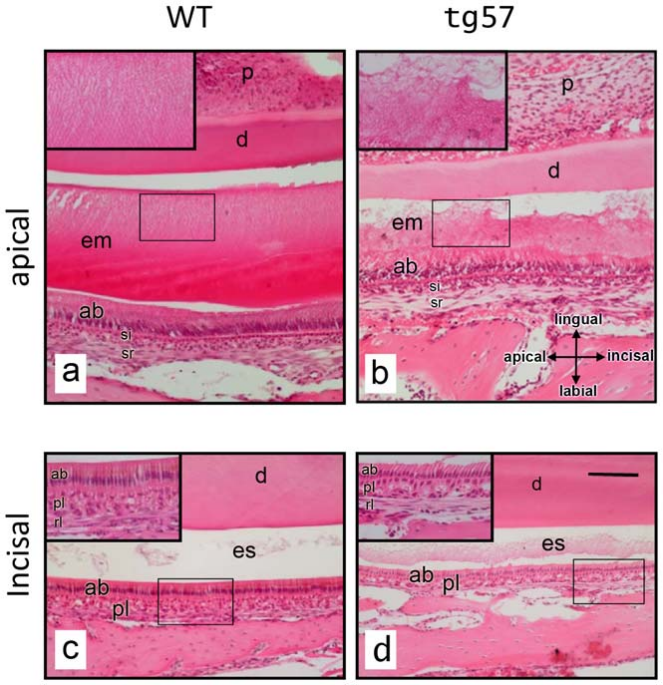
Histological analysis. Sagittal sections of mandibular incisors of wild type (WT) and transgenic (tg57) mice were stained with hematoxylin and eosin. Comparable anatomical regions from apical (late secretory stage; a, b) and incisal (maturation stage; c, d) portions along the apical/incisal axis are shown and magnified in inserts. At the late secretory stage, the eosinophilic enamel matrix appears completely disorganized and thinner in tg57 animals, compared to WT, where an organized, striated pattern is visible. At the maturation stage (c, d), tg57 animals display a thinner enamel space containing more residual organic matrix compared to WT animals. The orientation that applies to all sections is indicated in (b). Abbreviations: ab-ameloblast layer; d-dentin; em-enamel matrix; es-enamel space; p-pulp; pl-papillary layer; rl-reticular layer; si-stratum intermedium; sr-stellate reticulum. Scale bar in (d): 200 µm.

The histological analysis of hematoxylin and eosin (H&E) stained sagittal incisor sections of mice at P30 revealed significant differences in the structural arrangement of the organic extracellular enamel matrix in the apical portion of the tooth, corresponding to the early maturation stage at the anatomical level of the second molar (Figure 6 a, b). While the eosin-stained enamel matrix in wild type animals showed a striated pattern indicating the presence of organized, interwoven structures typical of prismatic enamel (Fig. 6a, insert), the matrix in line 57 animals was much thinner and completely unstructured (Fig. 6b, insert). The enamel matrix at this anatomical location also appeared to contain less organic material in transgenic animals. Dental pulp, dentin, stratum intermedium, stellate reticulum and alveolar bone structures were not significantly altered in transgenic mice. Ameloblast cell bodies and nuclei aligned similarly in both wild type and transgenic animals, but the normally straight demarcation between the apical membrane of ameloblasts and the enamel matrix was highly irregular in tg57 animals (Fig. 6b). Closer to the tip of the incisor, at an anatomical location between the mesial side of the first molar and the alveolar bone margin (Fig. 6c, d), only little eosinophilic matrix was left behind after decalcification in wild type animals, while in line 57 animals the enamel space contained much more such residual organic material. The thickness of the enamel space was clearly reduced in transgenic animals, but the morphology of ameloblasts appeared unaltered and the underlying papillary and reticular layers that form from stratum intermedium and stellate reticulum were indistinguishable between wild type and transgenic mice. The apical surface of ameloblasts, however, showed discontinuities in tg57 animals with occasionally disconnected adjacent cells (Fig. 6c and d, inserts).

### Scanning Electron Microscopy

**Figure 7:**
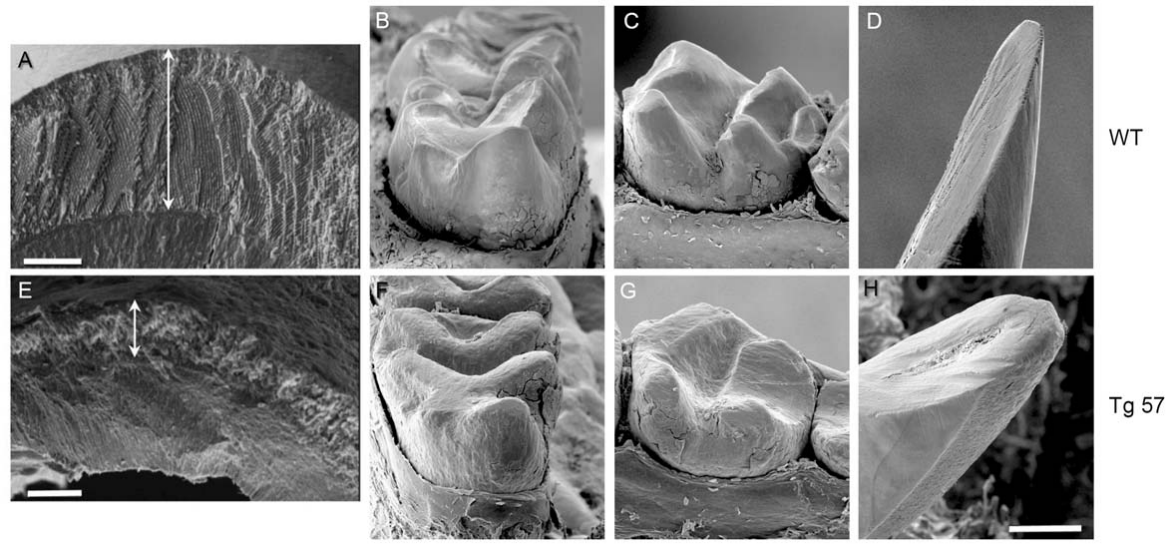
SEM analyses of teeth from wild type (A–D) and tg57 (E–H) animals. Images A and E show incisors fractured in cross section at the same anatomical region, approximately half way between the incisal edge and the gingival margin of the labial surface. Double-headed arrows indicate the substanital difference in enamel thickness. Enamel from wild type animals also showed the typical decussation pattern with a smooth enamel surface (A), while enamel in tg57 animals showed no decussation, and the surface was irregular and pitted (E). We also imaged whole teeth of WT and tg57 animals at 4 months of age, which revealed severe attrition of mandibular first molars (B, F; M1, mesial aspect), second molars (C, G; M2, lingual aspect) and incisors (D, H; buccal aspect) due to the loss of functional enamel. The positions of individual molar cusps, which are attrited in transgenic samples, are indicated by asterisks in wild type teeth. Scale bars: A and E: 50 µm; H (applicable to all other panels): 300 µm.

**Figure 8:**
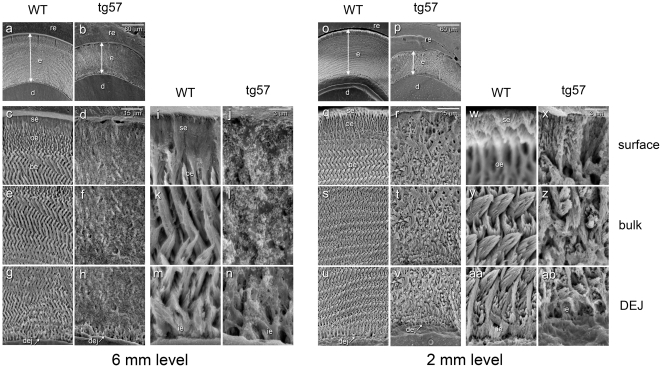
SEM analysis of transverse, etched enamel sections from wild type (WT) and transgenic (tg57) mice. Sections prepared at a distance of 6 mm (left) and 2 mm (right) from the incisal edge were imaged at various magnifications. At the 6 mm level, corresponding to the late maturation stage, enamel thickness was reduced by approximately 50% in transgenic animals (a, b). Higher magnification revealed the irregular enamel structure and residual organic matrix in transgenic mice at the enamel surface (c,d and i, j) and the bulk enamel (e, f and k, l). In contrast, the structure of the aprismatic initial enamel (ie) layer at the dentin-enamel junction (dej) was similar between wild type and tg57 animals (m, n). At the 2 mm level, where enamel is fully mature, its thickness in tg57 animals (p) was reduced to approximately 30% of that in wild type littermates (o), and higher magnification showed dramatic structural differences at the surface (q, r and w, x) and bulk (s, t and y, z) enamel layers. The enamel crystal structure at the initial enamel layer (ie in aa and ab) was again similar between WT and tg57 animals, but the transition to the regular prismatic enamel structure was not observed in tg57 animals. At the enamel surface (w, x) the discrete aprismatic surface layer of about 3–5 µm (se in w), which was more acid resistant than the underlying outer enamel layer as seen by the abrupt slope of acid-etched material, and the outer enamel layer featuring parallel enamel prisms (w) in wild type animals were replaced by a densely mineralized, compact layer in tg57 mice (x). Abbreviations: be, bulk enamel; d, dentin; dej, dentin-enamel junction; e, enamel; ie, inner enamel; oe, outer enamel; re, embedding resin; se, surface enamel; ep, enamel prism). The size of scale bars (a, b and o, p: 60 µm; c–h and q–v: 15 µm; i–n and w–ab: 3 µm) is indicated.

SEM analyses of incisors from 9 week-old mice, which were fractured at the level of the mesial surface of the first molar, revealed the typical enamel rod/interrod structure in wild type animals (Figure 7A) with normal decussation patters, producing a fairly even plane of fracture perpendicular to the long incisal axis. In contrast, the enamel in line 57 animals was much thinner (∼30% of wild type) and showed a highly irregular microstructure with no visible decussation patters. The outer enamel surface in wild type animals was generally smooth and hard, but the much thinner enamel in animals from lines 57 displayed an irregular and pitted outer surface, and was easily damaged during dissection (Fig. 7E). SEM imaging of teeth from 4 month-old wild type (Fig. 7B–D) and transgenic mice (Fig. 7F–H) revealed severe attrition in first and second molars with all cusps affected and in incisors, where attrition had progressed to the point of pulp exposure (Fig. 7H). More detailed analyses of the enamel microstructure in ground and etched transverse incisor sections at a distance of 6 and 2 mm from the incisal edge (Fig. 8) showed that the organized interdigitated arrangement of enamel prisms, which was observed in wild type animals at the 6 mm level, was severely disrupted in tg57 animals. Although the structure of the initial aprismatic enamel layer immediately adjacent to the DEJ was similar between WT and tg57 samples (Fig. 8m, n), the prismatic structure in bulk enamel (Fig. 8k) never forms in tg57 animals (Fig. 8l). Consistent with results from Fig. 6c and 6d, at an anatomical position similar to the 6 mm level shown in Fig. 8, the presence of residual organic matrix reduced the exposition of enamel prisms by phosphoric acid etching. At the erupted portion of the incisor (2 mm level), where enamel is fully matured, the regular pattern of interlocking enamel prisms was revealed in WT animals, but the arrangement of prisms in tg57 was completely disorganized (Fig. 8y, z). The initial enamel layer at the DEJ was again similar between WT and tg57 animals, but the transition to highly organized prismatic enamel, which is observed within ∼8 µm from the DEJ in WT animals, never occured in tg57 enamel (Fig. 8aa, ab). The appearance of the enamel surface layer, which is known to be more acid resistant than outer or bulk enamel [15], was also significantly altered in tg57 enamel (Fig. 8w, x)

### Transmission Electron Microscopy

**Figure 9:**
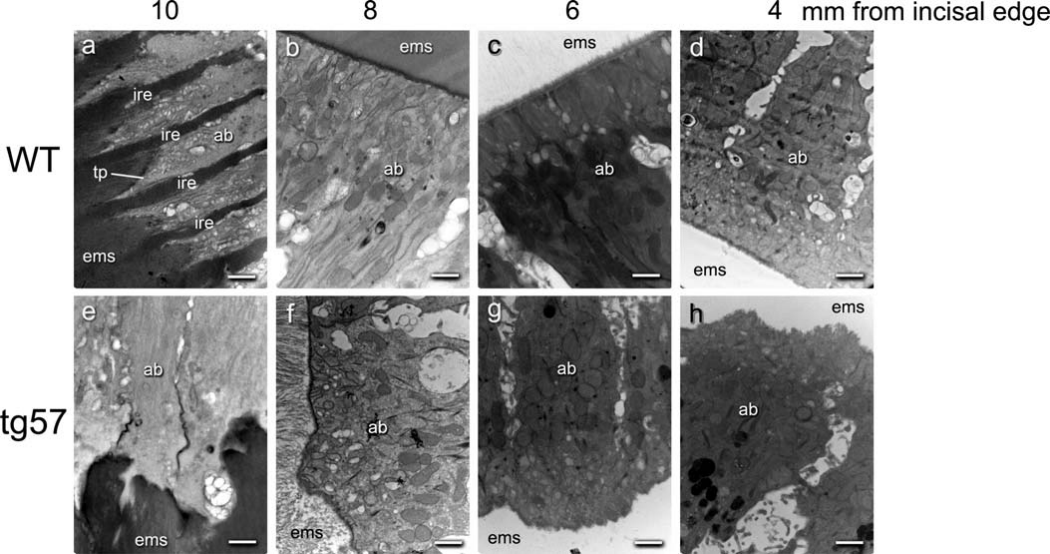
TEM analyses of the interface between the apical ameloblast surface and enamel matrix space (ems) at various positions along the apical-incisal axis in four week-old wild type (WT) and transgenic (tg57) animals. Images were taken from cross-sections made at a distance of 10, 8, 6 and 4 mm from the incisal edge of mandibular incisors. At the 10 mm level (a, e), corresponding to the secretory stage, the picket fence structure of orderly arranged Tomes' processes (tp), separated by interrod enamel (ire) is well developed in wild type animals, but absent in tg57 mice (e). The linear demarcation line between ameloblasts and the enamel space that forms after retraction of Tomes' process at the early (8 mm), mid (6 mm) and late (4 mm) maturation stages in wild type animals (b, c, d) appears highly irregular at all stages in transgenic animals (f, g, h). Abbreviations: ab, ameloblast; ems, enamel matrix space; tp, Tomes' process. Scale bars represent 500 nm.

Transmission electron microscopy studies were conducted to investigate the effect of TgAMTN overexpression on the cellular morphology of ameloblasts at various stages of enamel formation (Fig. 9). In wild type enamel, prominent Tomes' processes as hallmarks of secretory ameloblasts were found at the 10 mm level (Fig. 9a). During the early, mid and late maturation stages at 8, 6 and 4 mm levels, respectively, the apical surface of ameloblasts formed a linear interface with the enamel space (Fig. 9 b–d). In contrast, Tomes' process never formed in secretory stage ameloblasts of tg57 mice (Fig. 9e), and the compartmentalization of the enamel matrix into rod and interrod enamel [16] never occurred. During subsequent post-secretory stages of amelogenesis, the interface between the apical ameloblast surface and the enamel space maintained its irregular shape (Fig. 9f–h). No obvious differences were observed in the cellular structures of ameloblasts (secretory vesicles, pigment granules, Golgi, mitochondria, desmosomes, terminal webs) between wild type and transgenic animals.

### Mechanical hardness testing

Knoop's hardness of the enamel in wild type animals was found to be 2.2±0.4 GPa, consistent with previous data [17], but in tg57 animals the enamel was found to be too brittle to obtain any defined and reproducible indentation profiles. While it was thus impossible to derive values for enamel hardness, this result clearly indicates the severely compromised mechanical properties of enamel in the line 57 animals.

## Discussion

The purpose of this study was to generate transgenic mice that overexpress AMTN under the amelogenin promoter to investigate the effects on EMP expression, and ameloblast and hierarchical enamel structure. The *Amel* promoter has been shown to drive expression of several target genes in secretory stage and transition zone of ameloblasts [10], [11], [12], [13]. Considering that AMTN is predominantly expressed in maturation stage ameloblasts, such animals would not only overexpress, but also misexpress TgAMTN at an earlier stage than normal. Such overexpression allows for the analysis of AMTN effects during enamel growth. The rationale for incorporating the individual components of the transgenic construct has been detailed elsewhere [12]. In this report we have used three repeats of the FLAG epitope in frame with and located at the N-terminus of the AMTN sequence to detect transgene expression by Western blot and immunohistochemistry. We have created two different transgenic lines (57 and 457), which show comparable genomic copy numbers based on Southern blot analysis, and protein levels based on Western blot analysis. For reasons of space constraint we provide data for one representative line (line 57) only; animals from line 457 produced identical results to those presented here for line 57. Previous reports using similar constructs for the overexpression of other enamel genes have produced, compared and analyzed several transgenic lines [10], [11], [12], [13]. Most of them have not found any significant dose-dependent effects of transgene overexpression with the exception of transgenic mice overexpressing tuftelin, where an increased disruption to the rod/interrod architecture was observed in parallel with higher tuftelin expression levels [10] and mice overexpressing dentin phosphoproteins (Dpp), where increasing levels of transgene expression led to increasingly severe enamel abnormalities [11]. In our analysis, two lines with similar transgene copy numbers were obtained, although the absolute number of transgene copies has not been determined. The incorporation of the transgene at similar levels also resulted in correspondingly similar protein expression levels as demonstrated by Western blot. The AMTN signal in Western blots appeared as double bands when using both anti-Flag and anti-Amtn antibodies (Fig. 3). The lower molecular weight band is likely a product of proteolytic processing, as it appeared in the recombinant murine AMTN protein only after storage at 4°C for several weeks. The expected size of the transgenically produced protein is 22.4 kDa, and the observed signals with both Flag and Amtn antibodies correspond well with this size, indicating that the protein is not post-translationally modified to any significant degree. The appearance of only two main immunoreactive bands for the transgenically produced protein at the theoretically expected molecular weight also indicates that AMTN is not subjected to extensive proteolytic degradation by enamel proteases such as MMP20 and KLK4 [18], although the structural differences between both immunoreactive bands remain to be determined. Immunohistochemistry experiments clearly confirmed the overexpression of AMTN, although AMTN signal intensities were somewhat variable between different histological preparations. This is likely due to subtle differences in tissue manipulation and level of sectioning, inherent to immunohistochemical experimentation.

Significant differences in qualitative expression patterns at various stages of amelogenesis in molars and incisors (secretory, transition and maturation stages) between wild type and transgenic animals were obvious for AMTN only (Fig. 4 and 5). Prior to this study, the different expression profile of AMEL in secretory stage ameloblasts and AMTN mainly in maturation stage ameloblasts suggested the possibility that AMTN might act as a signal to terminate AMEL expression during amelogenesis. However, the expression patterns of EMPs (AMEL, AMBN) and ODAM were found to be essentially unaffected by TgAMTN overexpression, indicating that AMTN does not have a direct effect on the expression of these proteins. Rather, the observed effect on the structural architecture of the enamel matrix appears to be the result of a direct effect of AMTN, which, when increased in the model presented here, likely alters the structural organization of the organic matrix that provides instructive information for controlled mineralization. Whether this effect is the result of a direct binding of AMTN to mineral, or to other EMPs, remains to be determined. Since the primary sequence of AMTN contains only limited amounts of acidic amino acids, which have been shown to mediate hydroxyapatite binding in other mineral-associated proteins [19], it will be particularly important to determine whether AMTN binds to other extracellular matrix proteins that are expressed during, and affect, enamel formation. The fact that other proteins have been expressed at high levels from similar constructs in ameloblasts of transgenic mice, and as a result show no obvious alterations of the enamel structure [13], suggests that the observed effects are specific to AMTN.

The molecular mechanisms that control the structural transition from the typical rod/interrod structure of bulk enamel to the more compact, aprismatic layer of last-formed surface (“final”) enamel are not well understood. Since current studies have localized AMTN predominantly at the enamel/ameloblast interface in incisors of rats [7] and mice [4] it is conceivable that AMTN, possibly in co-operation with ODAM, is involved in the structural transition from bulk to surface enamel, promoting the formation of the compact enamel surface layer. If this is indeed the case then the overexpression of AMTN at a stage earlier than normal would be expected to result in structural alterations that resemble those observed at the outer and surface enamel layers. The formation of these final enamel layers coincides spatially and temporally with the retraction of Tomes' process. Indeed, the irregular structure of enamel that forms in the presence of TgAMTN supports a role for amelotin in terminating the regular arrangement of hydroxyapatite prisms. The clearly reduced enamel thickness in line 57 animals may also support a functional role for AMTN in disrupting the longitudinal enamel prism growth. In wild type rodent incisors, the formation of the aprismatic enamel surface coincides with the disappearance of Tomes' process in ameloblasts at the transition stage [8] and the expression of AMTN increases dramatically at the same location [4], [7]. Although Tomes' process is a critical hallmark of secretory stage ameloblasts, little is known about the molecular mechanisms that regulate its formation, function and retraction. This lack of knowledge is largely due to the unavailability of in vitro model systems that display – and allow study of - this anatomical feature of ameloblasts. It is known, however, that during the formation of initial enamel at the dentin-enamel junction (DEJ) the distal portion of Tomes' process does not yet exist, and only appears when the typical bulk enamel structure with rod and interrod compartments forms [20]. We thus speculate that the presence of AMTN at the initial stage of enamel formation disrupts the development of such an organized rod/interrod structure (as seen in Fig. 8), and leads to the failure of Tomes' process to develop (Fig. 9 a, e). The absence of Tomes' process is thus likely a consequence of disturbed crystal growth at interrod areas, which normally lead to the “picket fence” appearance of the ameloblast/enamel interface at this stage. However, whether there is a causal relationship between AMTN expression and the loss of Tomes' process at the transition stage under normal conditions, and the nature of such a relationship, is beyond the scope of this contribution but opens interesting areas for future research.

In our current study we have shown that AMTN overexpression does not notably affect the expression of two EMPs, AMEL and AMBN, which are normally expressed during the secretory stage. In contrast, AMTN overexpression resulted in a slight increase of ODAM expression in secretory stage ameloblasts. AMEL and AMBN on one hand, and AMTN and ODAM on the other hand, are expressed during the early and late stage of amelogenesis, respectively. It thus appears that distinct proteins activities are responsible for the formation and maturation of enamel and that AMTN and ODAM expression may not be independent of each other. The effects of overexpression or disruption of any one particular enamel gene on the expression level(s) of other enamel genes have not been elucidated in detail, and we expect the transgenic model presented here to be a useful tool for such studies. It also remains to be determined whether the overexpression of AMTN affects the expression level, pattern or activity of enamel-related proteolytic enzymes such as KLK4 or MMP20 to ultimately affect enamel matrix dynamics. Some clues to the activity of AMTN might also be related to the identification of novel proteases involved in enamel development such as chymotrypsin-c (encoded by CTRC) [21], since the expression profiles of AMTN and CTRC are essentially identical. Based on its localization at the enamel/ameloblast interface [4] it is possible that AMTN may also play a role in modulating transport phenomena to and from the mineralized enamel to regulate pH, ion transport, and/or the removal of organic matrix from the maturing enamel.

While we are still in the process of understanding the specific individual and collective contributions of enamel matrix proteins to enamel biomineralization, we have demonstrated that AMTN has a profound effect on the structure and mechanical properties of this functional bioceramic material. It is to be expected that more detailed future analyses of its role in establishing the enamel microstructure will provide a more comprehensive picture of the mechanisms involved in enamel biomineralization. A complete understanding of this process is required to devise strategies to re-mineralize, regenerate or engineer natural dental enamel, and to meet the clinical demands for natural restorative biomaterials.

## Materials and Methods

### Plasmid construction

The mouse amelogenin promoter [22], [23] was used in the construction of a plasmid cassette to express the amelotin protein, and this plasmid has been used to produce transgenic mice. Mouse amelotin cDNA with appropriate restriction sites was prepared by PCR using the forward primer 5′- G GATATC TTA CCA AAG CAG CTT AAC CCT which included an EcoRV restriction site (underlined) and a reverse primer 5′- GCCTCATAAAGG TGA AAC AGC TTA CTG AGT TCT that include an EcoNI restriction site and the stop codon (both underlined). This amplified DNA product was cut with EcoRV and EcoNI and then inserted into a previously generated vector containing the mouse amelogenin promoter, intron 1, the amelogenin signal peptide, and 3 repeats of the FLAG epitope. The final plasmid construct (Figure 1) was sequenced through the entire open reading frame to ensure that there were no PCR or cloning errors. This plasmid construct and its translated product will be referred to as pTgAMTN and TgAMTN, respectively.

### Transgenic animal production and verification of transgene status

All animal manipulation was performed under approved protocols (University of Toronto AUP Nr. 20008384 and University of Southern California AUP Nr. 9666) and complied with institutional and federal guidelines. Transgenic mouse lines were prepared as described elsewhere [13], [22], [24], [25]. Briefly, fertilized eggs for microinjection were harvested from super-ovulated six-week old female F1(C57Bl/6J×CBA/J) mice impregnated by adult male F1(C57Bl/6J×CBA/J) mice. For embryo transfer, pseudo-pregnant females were produced by mating CD1 adult females with a vasectomized CD1 adult male. Microinjection of DNA and oviduct transfer of injected zygotes was performed as described [24] using the ∼5.6 kb Sma I/Bam HI fragment of the plasmid pTgAMTN (Figure 1). Two independent lines obtained from this microinjection experiment (numbers 57 and 457), both with a presumed unique site of DNA integration, were bred beyond three generations before conducting any analyses. Animals were assessed for transgene status by Southern blot hybridization of genomic DNA digested with PstI, and hybridized to random primed ^32^P-labeled Eco RV/Pst I 528 bp amelotin single-stranded cDNA. Subsequent genotyping analysis of animals was performed by PCR using 2 µl of genomic DNA solution prepared from tail tissue (DNeasy Tissue Kit, Qiagen) with Titanium Taq polymerase (Titanium Taq Kit, Clontech Inc.) and the primers F4 (5′-C TTT TGG TCC TCT AAC TCG TTA-3′; a forward primer located in amelogenin intron 1 region) and R3 (5′-GA AGC AGG GTT AAG CTG CTT-3′; a reverse primer located in the amelotin cDNA region) to generate an amplicon of 429 bp. PCR was performed in a final volume of 25 µl under the following conditions: 94°C/5 minutes, followed by 40 cycles of (94°C/45 sec; 65°C/45 sec; 72°C/90 sec) and a final extension at 72°C for 10 minutes.

### Western blotting

For Western blots using anti-FLAG and anti-GAPDH primary antibodies (Fig. 3A) the following procedure was applied: First and second mandibular molars were dissected from 3–4 day old pups using a standard dissecting stereoscopic microscope. Tissues were lysed in 2× SDS-PAGE loading buffer (50 µl per mouse) using a tissue homogenizer. The samples were boiled for five minutes, chilled on ice and centrifuged to remove residual debris. The protein lysate was resolved according to size on 12% SDS-PAGE (20 µl each) and a Western blot was performed using a monoclonal antibody recognizing the FLAG epitope DYKDDDDK (Sigma-Aldrich Anti-FLAG M2, peroxidase conjugated, catalogue # A8592) and a mouse anti-glyceraldehyde-3-phosphate dehydrogenase (anti-GAPDH; Millipore; catalogue #MAB374) with the Amersham ECL kit (GE Healthcare Bio-Sciences Corporation). Prior to Western blots using an anti-AMTN primary antibody (Fig. 3B) the amelotin protein was enriched by immunoprecipitation as follows: First and second mandibular molars from 3–4 day old pups were extracted with 0.5 M acetic acid and the acid extracts desalted over a Sephadex G-25 column (PD10, GE Healthcare, catalogue # 17-0851-01). Extracts were then incubated with protein G agarose (Invitrogen, Carlsbad,CA, USA, catalogue# 15920-010), centrifuged and the supernatant mixed with approximately 1 µg of an affinity-purified rabbit antibody against the full-length recombinant mouse amelotin protein (FL-rmAMTN). AMTN-immunoglobulin complexes were precipitated with protein G agarose, washed with PBS, eluted with SDS-PAGE loading buffer and subjected to SDS-PAGE as above. Western blotting was performed using the FL-rmAMTN primary antibody and a goat-anti-rabbit HRP-conjugated secondary antibody (Biorad, Mississauga, ON, Canada; catalogue # 170-6515) using the Immun-Star Western kit (Biorad, catalogue # 170-5070) according to the manufacturer's recommendations. Primary antibodies FL-rmAMTN, FLAG and GAPDH were used at a dilution of 1∶4,000; 1∶2,000; and 1∶200, respectively. For protein competition, the FL-rmAMTN antibody was incubated with a 200-fold molar excess of the rmAMTN protein at 4°C over night and cleared by centrifugation prior to use.

### Histology and Immunohistochemistry

Animals at 4 and 30 days of age from each genotype (wild type, line 57 and line 457) were sacrificed for this analysis to reflect pre-eruptive and mature stages of tooth development. Animals were euthanized by CO_2_ inhalation, mandibular arches dissected, freed from excess skin and soft tissue and separated along the midline. After fixation in 4% paraformaldehyde (PFA) in phosphate buffered saline (PBS) at 4°C over night hemimandibles were decalcified in 12.5% EDTA (pH 7.2) for three days (tissues from four day-old animals) or ten days (30 day-old animals) with daily solution changes, then embedded in paraffin. Six micrometer thick sagittal sections were prepared as described previously [2], [13], [26] and used for histological and immunohistochemical analyses (Envision+ kit HRP, DAB, DAKO Canada, Burlington, ON). Sections were either stained with hematoxylin/eosin (H&E) for general histological evaluation, or probed with a rabbit anti-FLAG antibody (catalogue # F7425; Sigma-Aldrich,; dilution 1∶3,000) to demonstrate tissue specific expression of the introduced transgene. In addition, primary rabbit antibodies for mouse amelotin [4], diluted 1∶500, amelogenin (Abcam Cat. # 59705; 1∶2000), amelin/ameloblastin [27]; (a gift from Dr. M. Wendel, Centre for Oral Biology, Huddinge, Sweden; 1∶10,000) and Odam [6]; (gift from Dr. A. Nanci, Université de Montréal, QC; 1∶10,000) were used. Following color development with diaminobenzidine (DAB), sections were counterstained with methyl green (DAKO Canada) and mounted in Biomeda™ Gel/Mount media (Electron Microscopy Sciences, Hatfield, PA, USA). Images were captured with a Pixelink (Ottawa, ON, Canada) digital camera (model PL-A623C) mounted on an Eclipse E400 microscope (Nikon Canada, Mississauga, ON).

### Scanning electron microscopy

All scanning electron microscopy (SEM) data was obtained from heterozygous animals. Age-matched non-transgenic animals from each animal line served as controls. Six-week old transgenic and non-transgenic animals were sacrificed by CO_2_ asphyxiation. For fractured surface studies (to reveal the untreated enamel structure) the lower incisors were extracted and air-dried. Each sample was mechanically fractured cross-sectionally at the same anatomical region of the crown, in the mature enamel. For studies on sectioned and etched samples, hemimandibles from 9 week-old mice were fixed in 4% paraformaldehyde in PBS buffer at 4°C over night and transverse slices were prepared at every 2 mm from the incisal tip using a low speed saw (Buehler IsoMET®, Whitby, ON, Canada) with a diamond wheel (Part # DWH4123, South Bay Technology, Inc., San Clemente, CA, USA). Segments were dehydrated with graded acetone for about 12 h each, 100% acetone O/N at RT, then embedded with epoxy resin (Low Viscosity Embedding Media Spurr's Kit®, Electron Microscopy Sciences, Hatfield, PA, USA). The embedded samples were polished and etched with 38% phosphoric acid etching gel (Etch Rite®, Pulpdent Corp., Watertown, MA, USA). Samples were mounted on SEM stubs and coated with platinum (SC515 SEM Coating System, Polaron; Quorum Technologies, Ashford, Kent, UK). SEM pictures were captured using the Quartz PCI-Image Management System (Quartz Imaging Corporation, Vancouver, BC, Canada) on a S-2500 SEM machine (Hitachi, Roslyn Heights, NY, USA) operating at 10 kV.

### Transmission Electron Microscopy

Nine week-old animals were sacrificed by cardiac perfusion with 4%PFA, 1% glutaraldehyde in 0.1 M sodium cacodylate, hemimandibles dissected and fixed with fresh fixative at 4°C over night. Samples were then rinsed in PBS at 4°C overnight and demineralized with 0.8% glutaraldehyde, 12.5% EDTA in PBS for 5 days with three solution changes per day. After the secondary fixation with 1%OsO_4_, 0.5%K_2_Cr_2_O_7_, 0.5%K_4_[Fe(CN)_6_]·3H_2_O at RT for 2 hours, we performed *en bloc* staining with freshly prepared 2% uranyl acetate at RT for 2 hours. Samples were dehydrated in graded concentrations of ethanol and propylene oxide, then infiltrated and embedded in Jembed 812® resin (Canemco & Marivac, Lakefield, QC, Canada). Sections were prepared with a diamond knife (DiATOME ultra45, Diatome AG, Biel, Switzerland) in an EM UC6-NT microtome (Leica Microsystems, Concord, ON, Canada). Selected sections of 800 nm thickness were stained with Epoxy Tissue Stain® (Electron Microscopy Sciences) for quality control. Sections of 100 nm thickness were then post-stained with 2% uranyl acetate and mounted onto TEM grids (SPI, West Chester, PA, USA) for TEM analysis. Images were captured on a Tecnai 20 Transmission Electron Microscope (Philips, Amsterdam, The Netherlands).

### Mechanical hardness testing

Microindentation techniques as previously described [28] were used to measure enamel hardness in 6-week old (42-day post-natal) animals, five line 57 mice and six wild type controls. Briefly, freshly extracted intact murine lower left incisor teeth, kept moist at all times, were mounted in slow-set epoxy resin and sequentially ground in cross-section to a 0.1 µm alumina finish using a semiautomatic polisher (Buehler). Loads of 50 g were used with dwell times of 20 seconds using a customized manually-operated Vickers microhardness tester. Indentations were made within the erupted incisal thirds of the teeth, excepting the incisal-most 1 mm, approximately half way between the DEJ and the facial enamel surface. Indentations were examined by light microscopy, using polarization, interference, light/dark field, and measurements were made using a digital micrometer. The mean of 6 indentations was used to calculate means and standard errors.
